# Aortic valve replacement with annular patch enlargement for a patient with Werner’s syndrome and severe aortic stenosis

**DOI:** 10.1186/s13019-020-01219-7

**Published:** 2020-07-17

**Authors:** Kazuma Handa, Shinya Fukui, Yukitoshi Shirakawa, Tomohiko Sakamoto, Mutsunori Kitahara, Yumi Kakizawa, Hiroyuki Nishi

**Affiliations:** Department of Cardiovascular Surgery, Osaka General Medical Center, 3-1-56 Bandai-higashi, Sumiyoshi, Osaka, 556-8558 Japan

**Keywords:** Werner’s syndrome, Aortic stenosis, Small aortic annulus, Annular patch enlargement, Aortic valve replacement

## Abstract

**Background:**

Werner’s syndrome is an autosomal recessive rare genetic disorder characterized by clinical features suggestive of accelerated aging caused by mutation of the WRN gene. Although some reports exist of aortic valve replacement for aortic stenosis in patients with Werner’s syndrome, case using annular patch enlargement for a small aortic annulus are rare. We report herein the rare case of a patient with Werner’s syndrome and severe aortic stenosis treated by aortic valve replacement with annular patch enlargement.

**Case presentation:**

A 55-year-old woman genetically diagnosed with Werner’s syndrome suffered from symptomatic severe aortic stenosis with small annulus. Elective aortic valve replacement was performed. Intraoperatively the aortic annulus measured < 16 mm in diameter. Nicks technique for aortic root enlargement using a Hemashield patch was performed and an 18-mm mechanical valve was successfully inserted. After being discharged home her postoperative course was satisfactory for 2 years.

**Conclusions:**

Aortic valve replacement with annular patch enlargement to treat a small aortic annulus in a patient with Werner’s syndrome was successful. Treatment strategy must be determined while considering of the patient’s age, physical status, and severity of complications.

## Background

Werner’s syndrome (WS) is an autosomal recessive rare genetic disorder characterized by clinical features suggestive of accelerated aging caused by mutation of the WRN gene. WS has an average life expectancy of 54 years, attributable to cancer and arteriosclerotic disease including ischemic and valvular heart disease [1]. There have been some reports of aortic valve replacement (AVR) for aortic stenosis (AS) in patients with WS, but reported case using annular patch enlargement for a small aortic annulus are rare. Here, we report the successful employment of AVR with annular patch enlargement for an AS patient with WS.

## Case presentation

**Fig. 1:**
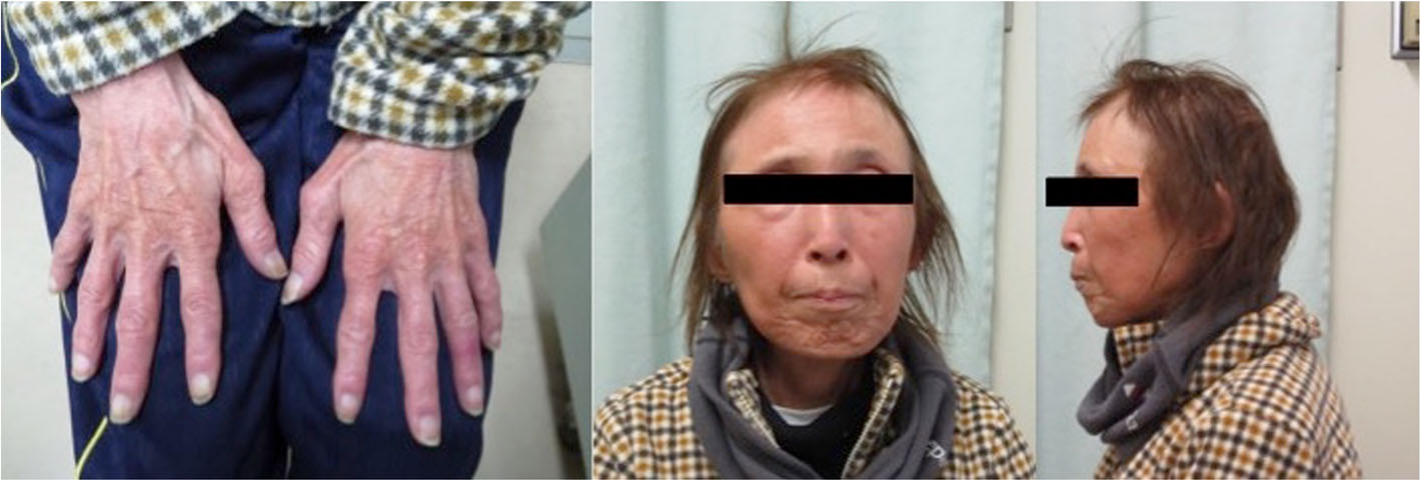
The patient’s physical characteristics, with graying and loss of hair, scleraderma-like skin, and “bird-like” face

A 55-year-old woman who suffered from dyspnea on effort as a result of severe AS was referred to our department for surgical treatment. She had a past medical history of osteoporosis, femoral neck fracture, bilateral cataract, uterine fibroid, and hypertension. She presented with short stature (140 cm), low weight (31 kg), high-pitched hoarse voice, graying and loss of hair, and scleroderma-like skin, with a “bird-like” face (Fig. 1). She was genetically diagnosed with WS and mutation of the WRN gene. Preoperative echocardiography showed severe AS with aortic valve area of 0.5 cm^2^, mean transaortic pressure gradient of 44 mmHg, calcification of aortic valves, and a small aortic annulus with a diameter of less than 18 mm. Coronary angiography showed a distal left posterolateral branch with a stenosis of 90%, which needed no intervention. Considering the options for treatment, namely transcatheter aortic valve implantation (TAVI) and surgical AVR using a bioprosthesis or mechanical valve, we scheduled AVR by mechanical valve because of her young age, normal cognitive function, and intact physical status. Following median full sternotomy, cardiopulmonary bypass was routinely established and aortotomy was undertaken under cardioplegic arrest. As the intraoperative findings, the tissue of the aortic valve, the aortic annulus, and the aorta were fragile with arteriosclerotic calcification. The aortic annular calcifications were presented at the non-coronary cusp and the right-coronary cusp. The aortic leaflets were thickening, but there was less calcification of the aortic leaflet. On intraoperative measurement, the aortic annulus was too small for the 16-mm ATS Open Pivot AP360 (ATS Medical, Minneapolis, MN, USA). A Nicks technique for the aortic annular enlargement using a teardrop-shaped Hemashield patch with a 5–0 Prolene (Ethicon, Somerville, NJ) running suture, enabled implantation of an 18-mm ATS Open Pivot AP360 (ATS Medical) at supra annular position with 12 pairs of interrupted non-everting mattress suture with a pledgeted 2–0 Tefdesser II (Kono Seisakusyo,Chiba, Japan). The aortotomy was closed by a 4–0 Prolene (Ethicon, Somerville, NJ) running suture reinforced with ePTFE felt strip. The patient had a prolonged recovery because of dysphagia despite extubation on the after the operation, and she was discharged for rehabilitation 28 days after the surgery. Thereafter she was discharged home and is now an outpatient of our department with no other postoperative complications 2 years after surgery.

## Discussion

Patients with WS have average life expectancy of 54 years [1], 7 years older than the median age of death reported in 1996, likely owing to improvements in medical care for both cancer and arteriosclerotic disease including ischemic and valvular heart disease [1]. The characteristic physical features of WS are aged appearance that includes skin atrophy, deep ulcerations around Achilles tendons and elbows, graying and loss of hair, bilateral cataracts, subcutaneous calcifications, high-pitched hoarse voice, and a “pinched” or “bird-like” facial appearance [1]. The clinical features include diabetes, hypogonadism, osteoporosis, sarcoma or thyroid carcinoma, arteriosclerosis, and atherosclerosis [1]. In the current case, the patient had almost all physical and clinical features of WS as well as mutation of the WRN gene, leading us to a definitive diagnosis.

To date, few case reports about AVR including TAVI for WS patients with AS (Table 1) have been published [2–6]. There is no consensus about valve selection and procedures for conventional AVR or TAVI. Carrel et al. reported the case of a 66-year-old patient with chronic skin ulcer infected with coagulase-positive *Staphylococcus* who underwent AVR with homograft, thus avoiding the risk of prosthetic valve endocarditis after aortic root enlargement, with no annulus enlargement [2]. Similar to our case, two patients underwent AVR using a mechanical valve. Grubitzsch et al. selected a mechanical valve perhaps because the patient was young (18 years old) [3]. In the case of the 41-year-old patient with liver cirrhosis reported by Sogawa et al., a bioprosthetic valve was chosen in order to avoid gastrointestinal hemorrhage, but could not be applied because of a too narrow aortic annulus and sinus of Valsalva. On considering that annular patch enlargement was too invasive for this cirrhosis patient, they reluctantly decided to use a mechanical valve after annular bougie instead of patch enlargement [4]. AVR using a bioprosthesis was reported by Ashida et al. in a 29-year-old woman with suspected WS via physical examination, whereby there was no need for annulus enlargement [5]. TAVI for a 51-year-old WS patient was reported by Masada et al. after considering the high risk for surgical AVR because of the patient’s frailty (wheelchair bound) and multiple ulcers in the extremities,

**Table 1:** Previous case reports of AVR or TAVI for WS patients

Author	Year	Age・Sex	Operation	Additional operation	Prosthetic valve	End point
Carrel T, et al. [2]	1994	66・Male	AVR	MVP, CABG× 1, Aortic root enlargement	Homograft	Home
Grubitzsch H, et al. [3]	2000	18・Female	AVR	MVR	Mechanical	Rehabilitation
Sogawa M, et al. [4]	2001	41・Male	AVR	Annular bougie	Mechanical (19 mm)	Home
Ashida T, et al. [5]	2005	29・Female	AVR	MVR (Biological・25 mm)	Biological (19 mm)	Home
Masada K, et al. [6]	2017	51・Male	TAVI		CoreValve (29 mm)	Home
Current case	2019	55・Female	AVR	Annular patch enlargement	Mechanical (18 mm)	Rehabilitation

*AVR* aortic valve replacement, *TAVI* transcatheter aortic valve implantation, *MVP* mitral valve plasty, *MVR* mitral valve replacement, *CABG* coronary artery bypass graft

In the current case, the patient was young and had no malignant diseases. In addition, her complications were stable. We determined that she would live longer after conventional AVR with a mechanical valve. To the best of our knowledge, this is the first case of AVR with annular patch enlargement for a small aortic annulus in a patient with WS. WS patients may have small aortic annuli [4]. Our patient’s annulus was too small for implantation of the prosthetic valve. Therefore, we performed a Nicks procedure with a Hemashield patch to enlarge the annulus. Because dementia is not a feature of WS, compliance with intake of warfarin would not be an issue if the mechanical valve was selected. Although the use of TAVI has spread worldwide, because our patient was completely independent in her daily life and her complications were not severe, we chose surgical AVR over TAVI. It is important that the cardiac team should collectively decide on the treatment approach, whether AVR or TAVI, and the valve selection for WS patients, always taking the patient’s life expectancy into consideration. Our strategy of surgical AVR for AS using a mechanical valve in a young, independent WS patient with stable complications is a feasible option.

## Conclusions

We report the successful use of AVR with annular patch enlargement for a small aortic annulus in a patient with WS. It is important to determine the valve selection and procedure at the time of AVR or TAVI while considering the average life expectancy, age, physical status, and severity of complications of the WS patient.

## Data Availability

The authors declare that all data in this article are available within this published article.

## Abbreviations

WS: Werner’s syndrome
AVR: Aortic valve replacement
AS: Aortic stenosis
TAVI: Transcatheter aortic valve implantation
